# Role of Microglia, Decreased Neurogenesis and Oligodendrocyte Depletion in Long COVID-Mediated Brain Impairments

**DOI:** 10.14336/AD.2023.10918

**Published:** 2023-12-01

**Authors:** Zhuang-Yao D. Wei, Ketty Liang, Ashok K. Shetty

**Affiliations:** ^1^Institute for Regenerative Medicine, Department of Cell Biology and Genetics, Texas A&M University Health Science Center School of Medicine, College Station, TX, USA.; ^2^Sam Houston State University College of Osteopathic Medicine, Conroe, TX, USA.

**Keywords:** Cognitive dysfunction, Long COVID, Neurological complications, Neuropathological changes, Neuroinflammation, SARS-COV-2, Systemic inflammation

## Abstract

Severe acute respiratory syndrome coronavirus 2 (SARS-CoV-2) is the cause of a recent worldwide coronavirus disease-2019 (COVID-19) pandemic. SARS-CoV-2 primarily causes an acute respiratory infection but can progress into significant neurological complications in some. Moreover, patients with severe acute COVID-19 could develop debilitating long-term sequela. Long-COVID is characterized by chronic symptoms that persist months after the initial infection. Common complaints are fatigue, myalgias, depression, anxiety, and “brain fog,” or cognitive and memory impairments. A recent study demonstrated that a mild COVID-19 respiratory infection could generate elevated proinflammatory cytokines and chemokines in the cerebral spinal fluid. This commentary discusses findings from this study, demonstrating that even a mild respiratory SARS-CoV-2 infection can cause considerable neuroinflammation with microglial and macrophage reactivity. Such changes could also be gleaned by measuring chemokines and cytokines in the circulating blood. Moreover, neuroinflammation caused by mild SARS-CoV-2 infection can also impair hippocampal neurogenesis, deplete oligodendrocytes, and decrease myelinated axons. All these changes likely contribute to cognitive deficits in long-COVID syndrome. Therefore, strategies capable of restraining neuroinflammation, maintaining better hippocampal neurogenesis, and preserving oligodendrocyte lineage differentiation and maturation may prevent or reduce the incidence of long-COVID after SARS-CoV-2 respiratory infection.

## Introduction

The recent worldwide pandemic, coronavirus disease-2019 (COVID-19), is caused by severe acute respiratory syndrome coronavirus 2 (SARS-CoV-2) infection. COVID-19 was first documented in China in 2019, causing a pneumonia-like disease in a handful of patients [1]. COVID-19 is primarily characterized by an acute respiratory infection with fever, fatigue, dry cough, myalgia, and dyspnea, with less typical symptoms being headache, dizziness, and abdominal pain [2]. Current treatments involve symptomatic care with antipyretics, oxygen therapy, antiviral drugs such as interferon alpha and remdesivir, and adequate hydration [3]. A recent study found that the discharge rate of COVID-19 patients was 52%, and the fatality rate was 5% [4]. Commonly, recovery from SARS-CoV-2 infection occurs within 2 to 6 weeks without sequelae (www.who.int/srilanka/news/detail/16-10-2021-post-covid-19-condition). However, some people report persistent, recurrent, or new symptoms long after the initial COVID-19 infection, a syndrome known as long-COVID syndrome. The World Health Organization (WHO) defines long-COVID as new or persistent symptoms occurring three months after initial infection and lasting for at least two months without other probable cause (www.who.int/europe/news-room/fact-sheets/item/post-covid-19-condition).

A hypothesized route of long-COVID pathogenesis lies in the neuroinflammatory effects of COVID-19. In addition to its commonly known respiratory effects, COVID-19 has been shown to cause significant central nervous system (CNS) inflammation and dysregulation. Several routes of neuroinvasion by SARS-CoV-2 have been proposed, including direct hematogenous spread, entry through the neural pathway between the olfactory epithelium and olfactory bulb, and hypoxic injury [5]. This commentary investigates the neuropathogenesis of SARS-CoV-2 CNS infection, including neuroinvasion, microglial reactivity, hippocampal neurogenesis impairment, and myelin dysregulation following respiratory COVID-19.

## CNS infection and long-COVID

While SARS-CoV-2 primarily infects the respiratory system, mounting evidence demonstrates its ability to invade and infect the CNS, resulting in altered taste and smell, headache, and fatigue [5]. The virus uses angiotensin-converting enzyme 2 (ACE2) as a receptor, which is highly expressed in the respiratory system cells and at variable levels in neural cells [6]). One proposed mechanism of CNS invasion is entry through the olfactory bulb [7, 8, 9]. By inoculating mice intranasally with COVID-19 variants, Seehusen and colleagues demonstrated subsequent infection and neuro-inflammation. Notably, the infection within the CNS appeared to spread from the olfactory bulb independent of ACE2 expression [10]. Such a finding implies that SARS-CoV-2 can gain entry via other molecules or that subthreshold levels of ACE2 may still be sufficient for viral entry. In another study of inoculated rhesus monkeys, SARS-CoV-2-related proteins were found to be restricted to the olfactory pathway, implicating the olfactory connectome as the basis of CNS spread [11]. The potential route of olfactory spread implicates the communications between nasal lymphatics and subarachnoid space [12, 13]. Transmucosal and axonal ports of entry have been proposed by human postmortem analyses of the neural-mucosal olfactory interface, which visualized intact CoV particles and SARS-CoV-2 RNA in the olfactory mucosa and adjacent neuroanatomical areas receiving olfactory tract projections [8]. As a result of the direct infection or secondary inflammation, the olfactory involvement can lead to the prolonged chemosensory dysfunction reported in the acute and longstanding phenotypes of COVID-19 [14].

Another proposed mechanism is through possible SARS-CoV-2 infection-related disruptions in the blood-brain barrier [15-17]. Erickson and colleagues created viral mouse models of COVID-19 in which they showed that the virus could cross the blood-brain barrier via adsorptive transcytosis and cause significant neuroinflammation [18]. Investigations of CNS viral load provided insights into the observed neurologic manifestations. Human postmortem studies of COVID-19 brains localized SARS-CoV-2 RNA signaling to the entorhinal, inferior frontal, and dorsolateral prefrontal cortices, which showed higher infected cell count with neurodegenerative disease [19]. This invasion of cognitive centers may explain exacerbated cognitive impairment in individuals with neurodegenerative disorders, including Alzheimer’s disease. Once infection of the CNS occurs, patients can have acute neurological and psychiatric symptoms such as headache, dizziness, anosmia, and impaired consciousness as some of the more common symptoms [20]. These neurological and psychiatric manifestations can be devastating and lead to long-lasting sequela like long-COVID [21]. Patients with long-COVID syndrome frequently complain of fatigue, palpitations, and difficulty focusing. These symptoms are long-lasting, impairing their daily lives [22].

There is no agreed-upon mechanism that causes long-COVID syndrome. However, some proposed mechanisms include multi-organ dysfunction from prolonged inflammation [23], enduring CNS hypoxia [24], and brainstem dysfunction [25]. Some other recently proposed mechanisms include reservoirs of COVID-19 lasting in tissues, immune dysregulation that can lead to reactivation of certain herpesviruses, and COVID-19 impacting the microbiota [26]. The diagnosis of long-COVID syndrome proves difficult as no objective studies can be done clinically. Diagnosis is currently made with careful documentation of symptoms and recognizing that the patient is exhibiting signs of long-COVID syndrome. However, such an approach proves difficult as symptoms are often non-descript, such as fatigue, arthralgias, myalgias, and chest pain [27].

Once diagnosed, treatments for long-COVID revolve around reducing fatigue and neuroinflammation. Light aerobic exercises have been recommended to strengthen respiratory muscles with complementary behavioral modification and psychological support. No specific pharmaceutical treatments are available to treat or alleviate long-COVID symptoms. However, over-the-counter medications such as Tylenol and non-steroidal anti-inflammatory drugs (NSAIDs) have been used for comfort measures [28]. Some of the popular dietary supplements have promise to reduce neuroinflammation, including resveratrol [29], curcumin [30, 31], melatonin [32], glycyrrhizin [33], and Omega-3 fatty acids [34]. In addition, living a healthy life with good food choices and regular physical exercise has been shown to go a long way in helping patients return to a standard level of functioning [35]. Thus, studying COVID-19 manifestations in the CNS is vital, focusing on appropriate diagnosis of neuroinflammation and treatment. Further research needs to be done on how COVID-19 leads to long-COVID syndrome. Studies must also be conducted on the efficacy of non-invasive methods, such as studying the composition of brain-derived extracellular vesicles in the blood to detect neuroinflammation [32, 36] or stratify patients to determine their risk for developing long-COVID syndrome or dementia.

## Inflammatory signaling and microglial alterations in the brain after SARS-CoV-2 infection

**Figure 1.**
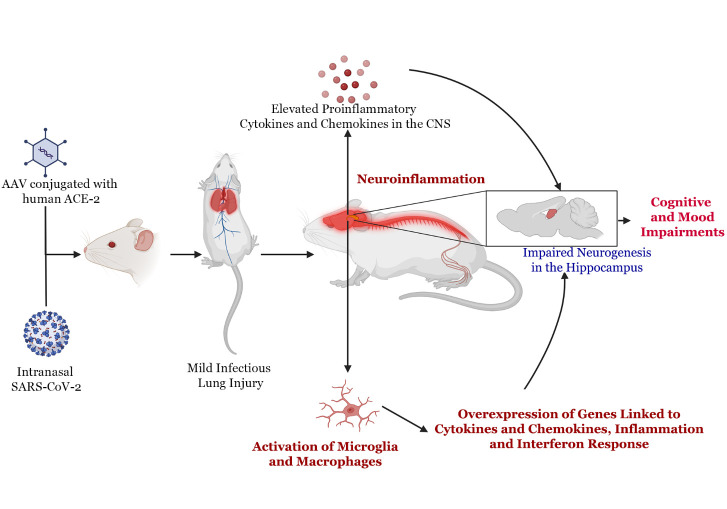
**Neuropathological sequelae in the mouse model after an intranasal administration of adeno-associated virus conjugated with human angiotensin converting enzyme 2 (ACE2) and severe acute respiratory syndrome coronavirus 2 (SARS-COV-2)**. Initially, such treatment results in mild infectious lung injury but leads to a signification elevation of multiple cytokines and chemokines in the brain with activation of microglia and macrophages. Such an inflammatory milieu impairs hippocampal function by reducing the extent of neurogenesis, which leads to memory and mood impairments.

Recently, a study by Fernandez-Castaneda and colleagues demonstrated several novel findings regarding CNS manifestations of COVID-19 [37]. In this study, induction of a mild respiratory COVID-19 in a mouse model led to neuroinflammation (Fig. 1). Mild respiratory COVID-19 was induced in a mouse model by first delivering human ACE2 conjugated to an adeno-associated vector (AAV) into the trachea and lungs, which was followed two weeks later by SARS-CoV-2 delivery through the intranasal route. Such an approach resulted in SARS-CoV-2 entry into the lungs with mild interstitial infiltrates and alveolar damage, but the virus did enter the brain. Interestingly, CNS changes were detected with upregulation of cytokines and chemokines in the cerebrospinal fluid (CSF) (Fig. 1). At seven days post-infection, interferon-gamma, interleukin-6 (IL-6), tumor necrosis factor-alpha (TNF-a), CXC motif chemokine ligand 10 (CXCL10), C-C motif chemokine ligand 7 (CCL7), CCL2, CCL11, granulocyte-macrophage colony-stimulating factor (GM-CSF) and B-cell activating factor (BAFF) were elevated. In contrast, CXCL10, CCL7, CCL2, CCL11, GMCSF, IL-10, and CCL5 were elevated at seven weeks post-infection. Notably, the CCL11 concentration in the CSF rose further at seven weeks in parallel to its rise in the circulating blood, which has significance because of its association with age-related cognitive impairment [38]. These findings suggest that CCL11 might be one of the underlying factors associated with brain fog experienced by patients with long-COVID, which is the experience of feeling mentally slow, spaced out, or unable to think or concentrate [39]. Long-COVID patients also perform poorly in standardized cognitive assessments, including attention, working memory, and executive function tasks. Elevated CCL11 levels, implicated in declining neurogenesis and impaired learning and memory, may explain the subjective and objective cognitive impairment experienced in long-COVID syndrome.

Furthermore, there was microglial and macrophage reactivity with increased CD68 expression in the mouse subcortical white matter but not in the cortical gray matter (Fig. 1). Investigation of postmortem brain tissues from 7 patients who either died suddenly or within days to weeks after COVID-19 symptom onset, also revealed microglial reactivity in the subcortical white matter, demonstrating that microglial activity occurs in humans as well [37]. Lung pathology revealed an infectious lung injury like that in the COVID-19 mouse model. Because of the heterogeneous nature of microglia, single-cell RNA sequencing was used on cortical and white matter microglia to identify their transcriptional profiles following SARS-CoV-2 infection. Further probing of reactive microglia via single-cell RNA sequencing revealed five states of the microglia, with three clusters overexpressing genes linked to chemokines, inflammation, and interferon response. Subpopulations of cells in these clusters showed elevated expressions of TNF-a, II1a, and II1b, implying robust inflammatory reactions. The other states of the microglia found include a homeostatic cluster and a cluster resembling axon tract microglia. The homeostatic cluster in post-COVID-19 also displayed increased expression of genes involved in inflammation and antigen processing and presentation. Chemokine and homeostatic gene signatures in post-COVID mice resembled a gene expression pattern displayed by disease-associated microglia in Alzheimer’s disease and white matter-associated microglia in aging [37]. Thus, even a mild COVID-19 in the mouse model can cause significant neuroinflammation with microglial reactivity and elevated cytokines and chemokines, akin to that observed in postmortem brain samples from COVID-19 patients [37]. A recent study employing immune-histochemical staining of autopsied brain tissues from COVID-19 patients revealed neuronal alterations and circulatory disturbances of microglia [40]. Another study demonstrated altered purinergic signaling in microglia via ADTP secretion production when exposed to SARS-CoV-2 spike protein. Purinergic signaling is a form of signaling that uses purine nucleotides to regulate cellular functions. These changes include upregulation and activation of purinergic receptors linked to neuroinflammatory events and neurogenerative diseases, notably P2Y6 and P2Y12. Such altered signaling has significance because multiple microglial purinergic receptors are implicated in neuroinflammatory events. The P2Y6 receptor stimulates phagocytosis after brain injury, which can lead to viable neuronal loss and memory impairment. Similarly, P2Y12 is associated with increased inducible nitric oxide synthase (iNOS) levels and inflammatory cytokines like IL-1β and TNF-α [41]. Furthermore, microgliosis has persisted even after viral clearance, contributing to cortical proteinopathies [42].

## COVID-19 and hippocampal neurogenesis

The hippocampus, a brain region vital for learning and memory function, is one of the few brain regions where neurogenesis continues throughout life in many species, including humans [43-48]. Hippocampal neurogenesis is one of the substrates supporting the encoding of new memories [49-52]. It also plays a role in mood function, as some of the effects of antidepressants are mediated through increased hippocampal neurogenesis [53]. The study by Fernández-Castañeda also suggested that mild COVID-19 can impair hippocampal function, as reactive microglia/macrophages found in the hippocampal white matter can adversely affect the various steps of neurogenesis (Fig. 1). Moreover, elevated levels of IL-6 and CCL11 observed in the CSF of post-COVID-19 mice can directly inhibit hippocampus neurogenesis [38, 54]. Villeda et al. demonstrated that increased CCL11 in mice can decrease hippocampal neurogenesis [38]. Furthermore, Parajuli et al. hypothesized that CCL11 promoted microglial migration and induced their production of reactive oxygen species - promoting neuronal death [55]. Such increased oxidative stress could be the potential mechanism by which CCL11 decreases neurogenesis. Moreover, IL-6 has been demonstrated to induce apoptosis of healthy adult hippocampal neural stem cells [56]. The impact of CCL11 was also evident from the study by Fernández-Castañeda and associates [37], in which systemic administration of CCL11 led to microglial/macrophage reactivity in the hippocampal white matter and reduced neurogenesis. Indeed, post-COVID-19 mice displayed decreased numbers of doublecortin+ newly born neurons in the subgranular zone-granule cell layer of the dentate gyrus at seven days post-infection, which persisted at seven weeks post-infection. Another study in hamsters has reported decreased hippocampal neurogenesis associated with microglial activation and upregulation of IL-1b and IL-6 [57]. These results suggest that SARS-CoV-2 infection-mediated microglial activation creates a proinflammatory microenvironment causing decreased neurogenesis. Further analyses of hippocampal neurons revealed morphological changes in pyramidal neurons, including shortened dendrites, decreased dendritic spines, reduced cellular branching, and altered neuronal distribution within pyramidal and granule cell layers [58]. Such interlinked alterations and reduced neuronal plasticity could contribute to memory impairments after COVID-19.

The implications of reduced hippocampal neurogenesis go beyond COVID-19. Studies in many animal models have demonstrated that physical and psychosocial stress results in decreased hippocampal neurogenesis, likely from mechanisms described above, leading to psychiatric disorders such as depression and anxiety [59]. Conversely, increasing hippocampal neurogenesis has been proposed as a novel mechanism by which antidepressant drugs improve mood function [60]. In addition, in R6/2 mice, a transgenic model of Huntington’s disease, neuropathological findings of decreased hippocampal neurogenesis have been observed [61]. Reduced neurogenesis of the hippocampus is also associated with Alzheimer’s Disease [62], Parkinson’s Disease [63], and epilepsy [44].

Fernández-Castañeda and colleagues also examined whether CCL11 concentration in the blood predicts cognitive impairment in long COVID-19 patients [37]. CCL11 concentration was increased in the circulating blood of 48 patients exhibiting long-COVID-related “brain fog” after mild SARS-CoV-2 infection, compared to 15 patients exhibiting long-COVID with no cognitive impairments. However, surprisingly, CCL11 concentration was higher in male patients. Still, the incidence of cognitive impairments and long-COVID was higher in female patients, implying that increased CCL11 concentration in the blood is not necessarily a sign of cognitive impairment.

## COVID-19, oligodendrocytes, and myelination

Myelination by oligodendrocytes plays a vital role in adaptive neurologic function by enhancing axonal conduction velocity and sustaining metabolic and trophic factors. With normal aging, the differentiation of oligodendrocyte precursor cells and myelin generation decreases, manifesting as declining cognitive function. In SARS-CoV-2 infection, reactive microglia can impair the generation of oligodendroglia lineage cells and myelin plasticity, resulting in a pathological presentation of cognitive function decline associated with long-COVID. The oligodendrocyte precursor cell population was unaffected at seven days post-infection but exhibited a mild decrease at seven weeks post-infection [37]. On the other hand, mature oligodendrocytes were depleted by ~33% at seven days, which persisted at seven weeks post-infection with significant myelin loss. The white matter areas also displayed decreased myelinated axon density at seven days post-infection, which did not recover at seven weeks post-infection. These changes in oligodendrocytes and myelin are similar to those seen in chemotherapy-related cognitive impairment, especially with methotrexate [37]. Chemotherapy-related cognitive impairment is also termed “chemo fog” and has many similarities with the “brain fog” seen in COVID-19 [64]. Myelin loss seen in the mild COVID-19 mouse model suggests that SARS-CoV-2 infection induces oligodendrocyte depletion, adversely impacting the myelination of axons and neural circuit function. Such changes likely contribute to neurodegenerative impairments in patients with long-COVID syndrome [37].

## Perspectives and future directions

Primary COVID-19 respiratory infection has been demonstrated to induce neuroinflammation via high viral load, cytokine storm, and multiple organ failure [65, 66]. The results of the study by Fernandez-Castaneda and colleagues are significant as they demonstrated that even mild SARS-CoV-2 respiratory infection in a mouse model could cause significant and persistent brain inflammation. The finding also raises the question of whether the degree of acute SARS-CoV-2 infection determines the severity of neurological complications. Some evidence supports the notion that the worse the respiratory insult, the more grave the hypoxia, and the more severe the resultant CNS inflammation [24]. However, a previous study suggested that decreased levels of CCL11 can increase stroke severity [67]. Additional studies are needed to determine the relationship between acute infection severity and the degree of CNS deficits. Nonetheless, CCL11 could serve as an objective measure to assess the risk of COVID-19 patients developing long-COVID syndrome or other complications of elevated neuroinflammation. Currently, the diagnosis of neuroinflammation in COVID-19 infection is complicated. From this perspective, human patients with long-COVID syndrome presenting elevated CCL11 in CSF and blood is of interest. Future studies need to assess the trajectory of CCL11 levels through longitudinal assessment during the acute or post-acute phases of COVID-19 infection. Such results help determine whether CCL11 could be employed as a biomarker for persistent neuroinflammation to predict possible long-COVID following respiratory SARS-Cov-2 infection. Also, the specificity of CCL11 to SARS-Cov-2 infection needs to be assessed as age-associated cognitive decline and chronic traumatic encephalopathy are associated with elevated CCL11 [68]. An elevated level of CCL11 is also seen in other pathological states such as asthma [69], head injuries [70], and alcohol dependence [71]. CCL11 levels can be used to stratify patients to assess the risk of their degree of neuroinflammation. For this to work, the degree of CCL11 elevation must correlate with the extent of neuroinflammation gleaned from additional measures, such as the analysis of brain-derived extracellular vesicles in the CSF and/or blood for proinflammatory cytokines such as TNF-a and IL-6, since both are typically upregulated after the respiratory SARS-Cov-2 infection-mediated brain inflammation.

Another benefit of tracking CCL11 concentration in the CSF, blood, and/or brain-derived extracellular vesicles is to gauge the extent of impairment in hippocampal neurogenesis and depletion of oligodendrocytes and axonal myelin sheath contributing to poor cognitive and memory function. The hippocampus is implicated in episodic and spatial memory, and neurogenesis in the dentate gyrus of the hippocampus plays a significant role in memory function, including pattern separation [72]. Impaired hippocampal neurogenesis can contribute to major depressive disorders and cognitive deficits associated with depression [73]. Oligodendrocyte depletion causing decreased myelination of axons can also contribute to the development of neurological and psychiatric disorders and dementia [74]. Impaired oligodendrocyte maturation and decreased myelin are implicated in schizophrenia, bipolar disorder, and Alzheimer’s [75]. Indeed, these can all play a role in the pathogenesis of long-COVID syndrome. There are some limitations to using CCL11 as a biomarker. As discussed, CCL11 may be elevated in other disease states such as Parkinson’s, Alzheimer’s, and Epilepsy. So, if CCL11 is used as a biomarker to monitor long-COVID, then any confounding medical problems should also be considered. CCL11 has also been reported to be elevated in prostate cancer [76] and asthma [69], both common diseases. Before CCL11 can be used as a biomarker, its specificity to long-COVID 19 should be determined.

In summary, the findings reported by Fernández-Castañeda and associates provide vital insights into the pathogenesis of long-COVID syndrome. The findings uncovered that significant neuroinflammation, typified by microglial and macrophage reactivity, can result from even a mild respiratory SARS-CoV-2 infection, which could be gleaned from elevated levels of CCL11 and other chemokines/cytokines in the CNS and the circulating blood. Furthermore, such inflammatory milieu also impaired hippocampal neurogenesis, depleted oligodendrocytes, and decreased myelinated axons, all of which can contribute to cognitive deficits in long-COVID syndrome. From these perspectives, strategies for preventing long-COVID after SARS-CoV-2 respiratory infection must focus on therapeutic measures capable of controlling neuroinflammation, maintaining better hippocampal neurogenesis, and preserving oligodendrocyte progenitors and their maturation. Apart from exploring promising drug therapy, commencing even a moderate physical exercise regimen after recovering from SARS-CoV-2 infection could halt the progression of pathological changes and long-COVID. Studies have shown moderate physical exercise can modulate proinflammatory microglia into noninflammatory or anti-inflammatory microglia [31]. Also, physical exercise can increase the concentrations of irisin and brain-derived neurotrophic factor, which could enhance hippocampal neurogenesis and oligodendrocyte maturation and improve myelin formation [32, 50, 77-79]. Such combinatorial effects of physical exercise can lead to better cognitive and mood function after recovering from COVID-19. In addition, the study provides evidence that CCL11 can be used as a potential biomarker for neuroinflammation found in long-COVID, as well as many other CNS pathologies that arise due to neuroinflammation. However, the specificity of elevated CCL11 levels must first be determined for each neurological disorder.
